# Long-term survival and healthcare utilization outcomes attributable to sepsis and pneumonia

**DOI:** 10.1186/1472-6963-12-432

**Published:** 2012-11-26

**Authors:** Andrew Dick, Hangsheng Liu, Jack Zwanziger, Eli Perencevich, E Yoko Furuya, Elaine Larson, Monika Pogorzelska-Maziarz, Patricia W Stone

**Affiliations:** 1 RAND Corporation, 20 Park Plaza. Suite 920, Boston, MA, 02116, USA; 2 University of Illinois, Chicago, 1603 W. Taylor Street, Chicago, IL, 60612, USA; 3 University of Iowa, Carver College of Medicine and Iowa City VA Health Care System, 601 Hwy 6 W 3E21, Iowa City, IA, 52246, USA; 4 Columbia University College of Physicians & Surgeons, NewYork-Presbyterian Hospital, 630. W. 168th Street, New York, NY, 10032, USA; 5 Columbia University, Mailman School of Public Health, School of Nursing, Center for Health Policy, 617 W. 168th Street, New York, NY, 10032, USA; 6 Columbia University, School of Nursing, Center for Health Policy 617 W. 168th Street, New York, NY, 10032, USA

**Keywords:** Community-associated infections, Sepsis, Pneumonia, Survival, Healthcare utilization

## Abstract

**Background:**

Hospital associated infections are major problems, which are increasing in incidence and very costly. However, most research has focused only on measuring consequences associated with the initial hospitalization. We explored the long-term consequences of infections in elderly Medicare patients admitted to an intensive care unit (ICU) and discharged alive, focusing on: sepsis, pneumonia, central-line-associated bloodstream infections (CLABSI), and ventilator-associated pneumonia (VAP); the relationships between the infections and long-term survival and resource utilization; and how resource utilization was related to impending death during the follow up period.

**Methods:**

Clinical data and one year pre- and five years post-index hospitalization Medicare records were examined. Hazard ratios (HR) and healthcare utilization incidence ratios (IR) were estimated from state of the art econometric models. Patient demographics (i.e., age, gender, race and health status) and Medicaid status (i.e., dual eligibility) were controlled for in these models.

**Results:**

In 17,537 patients, there were 1,062 sepsis, 1,802 pneumonia, 42 CLABSI and 52 VAP cases. These subjects accounted for 62,554 person-years post discharge. The sepsis and CLABSI cohorts were similar as were the pneumonia and VAP cohorts. Infection was associated with increased mortality (sepsis HR = 1.39, P < 0.01; and pneumonia HR = 1.58, P < 0.01) and the risk persisted throughout the follow-up period. Persons with sepsis and pneumonia experienced higher utilization than controls (e.g., IR for long-term care utilization for those with sepsis ranged from 2.67 to 1.93 in years 1 through 5); and, utilization was partially related to impending death.

**Conclusions:**

The infections had significant and lasting adverse consequences among the elderly. Yet, many of these infections may be preventable. Investments in infection prevention interventions are needed in both community and hospitals settings.

## Background

Pneumonia and sepsis are major problems, which are increasing in incidence, especially among the elderly [1-4]. Indeed, it has been estimated that approximately 10% of all intensive care unit (ICU) patients have severe sepsis on admission or during their ICU stay [5]. Further, despite technological advances in prevention, there has been no change in the relative risk of inpatient mortality from such infections in recent years [6]. Infections generate a substantial economic toll. For example, recently, in surgical patients the extra inpatient hospital costs of pneumonia and sepsis were estimated to be $32,900 and $46,400 respectively [7]. However, this research focused only on measuring the costs associated with the initial hospitalization.

There is increasing interest in the long-term outcomes associated with sepsis and pneumonia, although research in this area is limited [8,9]. In a recent systematic review, 23 studies examining mortality after hospital discharge with diagnosis of sepsis were found [10]; and, only two of the studies followed patients for up to five years and included measures of healthcare utilization [11,12]. The authors of the systematic review concluded patients with sepsis had ongoing mortality up to 2 years and they encouraged researchers to include longer-term endpoints. The evidence of the long-term consequences associated with pneumonia is even sparser. In a single-site study, researchers examined long-term mortality after hospitalization with pneumonia [13]. In this investigation, the researchers followed 457 patients after an index hospitalization for pneumonia and found poor long-term survival. We could identify no studies of the long-term clinical consequences of healthcare associated infections.

To assess the potential value of infection prevention initiatives in hospital and community settings within a business-case analysis framework, a better understanding of the long-term clinical and economic consequences of these infections is necessary [14]. Hence, the aim of this study was to examine in Medicare patients admitted to an ICU and discharged alive: the relationships between sepsis, pneumonia, central-line-associated bloodstream infections (CLABSI), and ventilator-associated pneumonia (VAP); the relationships between the infections and long-term survival and resource utilization; and to determine how much of the resource utilization was related to impending death during the follow up period.

## Methods

### Data sources

We analyzed a unique longitudinal national dataset initially created to study predictors of healthcare associated infections in elderly Medicare recipients during a hospitalization in an ICU [15,16]. In that study, 31 hospitals belonging to the Centers for Disease Control and Prevention’s (CDC) National Nosocomial Infections Surveillance system that had conducted device-associated infection surveillance in 2002 were recruited. We obtained Medicare claims data for the universe of elderly patients in the participating hospitals during the months that surveillance was conducted. This study was approved by the Institutional Review Boards at the Columbia University Medical Center and the RAND Corporation.

From this sample we defined “index ICU stays” as those that occurred during a period of CDC infection surveillance in 2002. An individual patient could contribute more than one index ICU stay to the sample as long as the additional ICU stay was in a month in which surveillance occurred. We merged these index ICU data with individual Medicare inpatient, outpatient and denominator data for the years 2001 through 2007, yielding a minimum of one year of data prior to, and up to five years of data following, the index ICU stay. All analyses that follow are conditional on being discharged alive from the index stay.

### Measures

#### Exposure

For each index ICU stay, we obtained sepsis and pneumonia status from the Medicare claims data and healthcare associated infections status from the CDC data. We defined exposure variables based on infection status during the index hospitalization including: 1) sepsis, 2) pneumonia, 3) CLABSI and 4) VAP. The fifth group was the control (i.e., none of these infections).

The definitions of sepsis and pneumonia were based on the International Classification of Diseases, Ninth Revision, Clinical Modification [ICD-9-CM] codes associated with the index ICU stay. Codes used for sepsis included ICD-9-CMs: 038 (septicemia), 995.91 (sepsis), 995.92 (severe sepsis), and 785.2 (septic shock), and for pneumonia, ICD-9-CMs: 482.0-482.2, 482.4-482.9 (pneumonia cases with a bacterial diagnosis code) [17]. These ICD-9-CM codes have been used in previous research and the 038 codes for septicemia and the 482 codes for pneumonia have been validated with a specificity and positive predictive value of 99% and 89%, and 99% and 85%, respectively [3,7,18].

Patients with either of the two device-associated infections of interest (CLABSI and VAP) were identified by the hospitals’ infection preventionists and reported into CDC’s system. All infection preventionists used the same direct surveillance protocols developed by the CDC and these protocols included both clinical and laboratory data [19]. In the infection groups, if a person was identified with a CLABSI, they were not included in the sepsis group, and similarly, if they were identified with a VAP they were not included in the pneumonia group. However, a patient could be identified in both the sepsis and pneumonia cohorts, or in both the VAP and CLABSI cohorts.

#### Outcomes

The principal outcome measures were survival and healthcare utilization. We used the date of death and the date of discharge from the index admission to define the length of survival. We used place and date of service to define healthcare utilization categories, including inpatient admissions, outpatient visits, emergency department admissions, long-term care admissions, and home healthcare visits. The broad *outpatient visits* category included office, outpatient hospital, ambulatory surgical center, federally qualified health center, state or local public health clinic, rural health clinic, and community mental health center visits. For each utilization category we generated annual counts (365-day period) for each of the five years following the index hospitalization discharge date.

#### Covariates

For each patient, both inpatient and outpatient Medicare data were used to generate measures of health status based on 30 aggregated condition codes and 184 hierarchical condition codes using the DxCG software [20-22]. These controls were calculated based on healthcare experiences during the year *prior* to the index admission. Our method used diagnostic information associated with prior hospitalizations, outpatient, and ambulatory services to characterize health status by considering multiple coexisting medical conditions and creating more aggregated groupings. The hierarchies served to 1) improve clinical validity (e.g., it is not useful to characterize a person with a more severe manifestation of diabetes the same as a less serious type); 2) overcome some of the limitations found in Medicare data due to coding practices (e.g., the proliferation of recorded diagnoses for the purpose of maximizing reimbursement); and 3) improve the precision of the risk adjustment [23,24]. We also included other patient demographics (age, gender, race) and Medicaid status (i.e., dual eligibility).

### Statistical analysis

For each subgroup, summary statistics were computed. Pearson’s Chi squared statistics or Fisher’s exact test statistics were used to test the equivalence of categorical variables. For continuous variables, t-tests or non-parametric tests were computed.

#### Mortality models

Patient survival outcomes across infection groups were examined first by calculating Kaplan-Meier survival functions and log-rank tests to detect differences among cohorts. We then estimated multiple variable Cox proportional hazard models to control for the effects of health status (aggregated condition codes and/or hierarchical condition codes measures) and other covariates. We tested the proportional hazard assumption by estimating alternative specifications that admitted interactions between analysis time and variables of interest. Finally, we estimated multiple variable parametric hazard models with continuous time frailty to account for unobserved heterogeneity in health status among subjects [25,26]. We considered alternative distributional assumptions for the parametric duration dependence (exponential, gamma, Gompertz, log logistic, log normal, and Weibull), selecting Weibull because of model fit and because it best matched the patterns of duration dependence identified by the Kaplan-Meier models. We considered gamma and inverse Gaussian distributions to model unobserved frailty, and selected the gamma distribution based on model performance.

#### Utilization models

The data were organized so that the unit of analysis was the person-year. We formulated multiple variable Poisson and negative binomial models for each healthcare utilization category; however, in the likelihood ratio test of over-dispersion the alphas were significantly different from zero, which led us to reject the Poisson models for the negative binomial models in every case.

Each subject could contribute up to five years of follow-up or until death. Subjects who died were at risk for healthcare utilization only for the part of the year in which they were alive. We defined an *exposure* variable to adjust these observations for the amount of time at risk during the year of death. Variation in time at risk is typically handled by including ln(*exposure)* as a variable in the model and restricting its coefficient to one, typically referred to as fitting exposure time as an offset. We refer to this specification as Model 1, which estimates the full relationship between infection and utilization but treats censoring (time at risk) as random.

Because the censoring (exposure) was a function of death, and since death is undoubtedly correlated with healthcare utilization, the censoring is unlikely to be random. To determine how much of the resource utilization was related to impending death during the follow up period, we admitted an additional polynomial to account for changes in utilization as a function of “time-until-death” and identify this specification as Model 2 (see Additional file 1 for full discussion). In Model 2, alternative specifications of the “time-until-death” included step, linear, quadratic, or third order functions. We also allowed for alternative periods for the “time-until-death” including 6 months, 12 months, or 24 months prior to death. The Model 2 specifications, therefore, separately identify an underlying pattern of utilization following discharge and an additive component based on the time until death following discharge. Huber-White sandwich estimators are used to calculate all standard errors [27,28].

## Results

Thirty-one hospitals from across the United States contributed data on 17,537 subjects who were discharged alive from 51 ICUs. The majority of the hospitals were large (median number of beds = 360), teaching (n = 24, 71%) hospitals, which was similar to the overall sample of CDC National Nosocomial Infections Surveillance system hospitals at the time.

Table 1 provides an overview of the sample, these subjects accounted for 62,554 person-years post discharge. There were 1,062 cases of sepsis, 1,802 cases of pneumonia, 42 cases of CLABSI and 52 cases of VAP. 309 patients were identified in both the sepsis and pneumonia groups; and 6 patients were identified in both the CLABSI and VAP groups. Because CLABSI and VAP were relatively rare, the associated multiple variable results are only reported in Additional file 2: Table S2.

**Table 1:** Sample characteristics

**Variables**	**No infection**	**Sepsis**		**Pneumonia**		**CLABSI**		**VAP**		**Between group difference**
	**(N = 14894)**	**(N = 1062)**		**(N = 1802)**		**(N = 42)**		**(N = 52)**		
Age (mean)	73.84	74.14		73.93		72.64		74.92		
Female (%)	48.60	49.90		50.30		57.10		36.50		
Race										
White (%)	88.80	86.60	^*^	87.60		90.50		88.50		
Black (%)	8.20	10.40	^*^	9.00		9.50		9.60		
Other (%)	3.10	3.00		3.40		0.00		1.90		
Dual Eligible (%)	16.30	21.30	^**^	23.10	^**^	21.40		11.50		^††^
No. ACCs (mean)	4.75	5.75	^**^	5.10	^**^	5.67	^**^	4.89		^††^
Days alive during follow-up (mean)	1246 1	869 3	^**^	877 3	^**^	883 8	^**^	937 1	^*^	^††^
Inpatient admissions (mean)										
Year 1	1.19	1.45	^**^	1.35	^**^	1.64		1.40		^††^
Year 2	0.87	1.10	^**^	1.13	^**^	0.88		0.63		^††^
Year 3	0.81	0.88		0.96	^**^	1.40		1.04		^†^
Year 4	0.78	0.80		0.93	^**^	0.94		0.68		
Year 5	0.73	0.74		0.86	^*^	0.50		0.86		
Outpatient visits (mean)										
Year 1	16.65	13.12	^**^	12.94	^**^	13.02		12.89		^††^
Year 2	15.85	13.87	^**^	14.30	^**^	12.96		13.10		^††^
Year 3	15.77	14.59		14.12	^**^	14.90		12.41		^††^
Year 4	15.49	14.49		13.70	^**^	12.47		12.46		^†^
Year 5	14.92	13.41		13.83		12.94		11.19		
Emergency Department visits (mean)										
Year 1	1.33	1.53	^*^	1.44		1.19		1.39		
Year 2	1.13	1.39		1.34	^*^	0.46		0.57		
Year 3	1.09	1.24		1.14		1.25		1.22		
Year 4	1.10	1.08		1.12		1.06		0.59		
Year 5	1.04	0.98		1.08		0.50		0.76		
Long-term care admissions (mean)										
Year 1	2.18	5.04	^**^	3.93	^**^	4.43		4.04		^††^
Year 2	1.62	3.89	^**^	3.17	^**^	2.58		2.13		^††^
Year 3	1.68	3.87	^**^	2.94	^**^	2.50		3.93		^††^
Year 4	1.88	3.39	^**^	3.76	^**^	3.35		4.18		^††^
Year 5	1.90	3.60	^**^	2.98	^**^	5.69	^*^	4.05		^††^
Home care visits (mean)										
Year 1	0.34	0.60	^**^	0.40		0.36		0.23		
Year 2	0.29	0.67	^**^	0.45		1.50	^*^	0.40		^††^
Year 3	0.26	0.60	^**^	0.47	^**^	0.60		0.11		^††^
Year 4	0.31	0.73	^**^	0.50	^*^	0.06		0.00		^††^
Year 5	0.30	0.64	^**^	0.39		0.00		0.10		

ACC – aggregate condition codes; Sepsis – community or healthcare-associated sepsis, excluding CLABSI; Pneumonia – community or healthcare-associated pneumonia, excluding VAP; CLABSI – central-line associated blood stream infection; VAP – ventilator associated pneumonia; Comparison between the control group and an infection group: * p < 0.05; **p < 0.01; Overall test between all groups: †p < 0.05; ††p < 0.01.

The mean age was 74 years, with no significant variation across infection groups. There were slightly more males than females in the sample but infection status was not associated with gender. The sample was largely white, with just over 8% black and 3% other race. Sepsis was more likely to occur in blacks than whites (P < 0.05), but race was not associated with other infection types. The sample was restricted to subjects who were covered by Medicare, but about 17% of the sample was also eligible for Medicaid (dual eligible). Dual eligibility was strongly associated with infection (P < 0.01), with these individuals over-represented in the sepsis and pneumonia groups. The mean number of aggregated condition codes during the year prior to the index ICU admission was nearly 5, with strong associations between the number of aggregated condition codes and infection status. Those with pneumonia or sepsis had significantly higher mean aggregated condition codes than those in the control group (all P values less than 0.01).

Survival following discharge from the index admission varied substantially among the different cohorts (P < 0.01); the mean number of days alive was substantially lower in each infection group (range 869 to 937 days) than in the control group (1246 days) with all P values less than 0.05. Diagnosis of sepsis and pneumonia were associated with increased inpatient admission, emergency care, long-term care, and home healthcare use, along with reduced rates of outpatient visits compared to no infection. For sepsis, the associations with long-term care and home healthcare were particularly large and lasting. The positive association between pneumonia and inpatient admissions was also persistent throughout the five-year study period.

### Survival models

**Figure 1:**
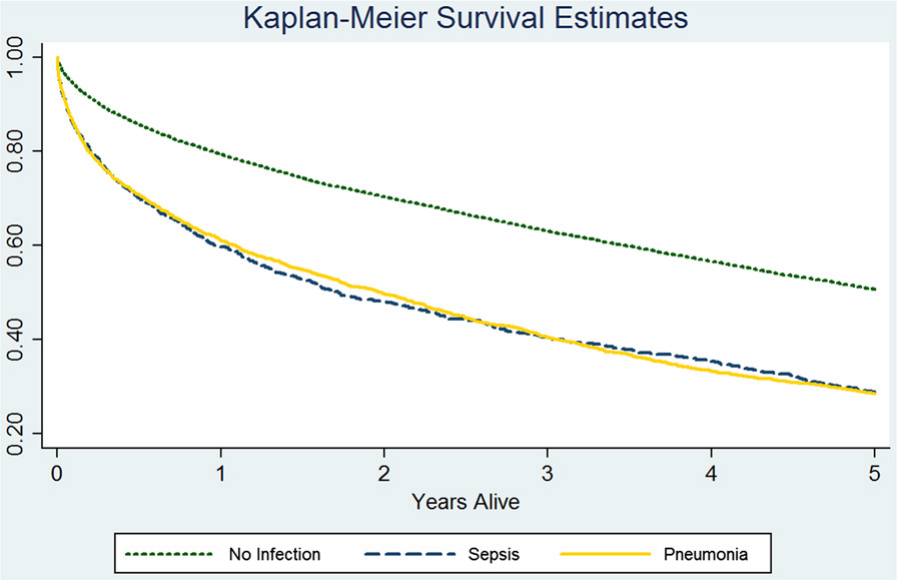
**Kaplan-Meier survival functions of three patient cohorts.** An overall log-rank test showed that the survivor functions differed across patient cohorts (p < 0.01); the survival of the no-infection group was longer than that of the sepsis group (p < 0.01) and the pneumonia group (p < 0.01).

Figure 1 presents a graph of the Kaplan-Meier survivor functions by group. The figure shows excess mortality associated with each infection category over the five-year period following discharge from the index admission. The log-rank tests indicated that: 1) overall survival differed by infection group (P < 0.01) and 2) in pair-wise comparisons, the control group differed from both sepsis and pneumonia groups (P < 0.01 in each case).

Table 2 presents the results of the multiple variable Cox proportional hazard and parametric frailty models. Tests of the proportional hazard assumption showed that the relationships of sepsis and pneumonia were not proportional. Thus, the models included interactions between sepsis and pneumonia and survival, distinguishing the first year post-discharge from the rest of the follow-up period. The results changed very little with alternative parametric specifications of either duration dependence or frailty (see Additional file 2: Table S2). Although unobserved frailty was present (P < 0.01), the results of the relationships between infection type and survival varied little in the two specifications, so we describe only results from the Weibull frailty model below. Infection was associated with increased mortality (sepsis HR = 1.39, P < 0.01; pneumonia HR = 1.58, P < 0.01). The relationships were similar for sepsis and CLABSI as well as pneumonia and VAP (see Additional file 2: Table S2). There was increased mortality risk associated with sepsis and pneumonia in the year following discharge (HR relative to other years = 1.17, P < 0.05 for pneumonia and 1.11, P = 0.11 for sepsis), and the risk persisted throughout the entire five-year follow-up period. The risk of dying among females was lower than among males (HR = 0.89, P < 0.01). There was no difference in survival by race, but there was a strong positive gradient with age. In addition, patients with dual eligibility had a greater risk of mortality (HR = 1.22, P < 0.01).

**Table 2:** Cox proportional hazard and frailty models, post discharge survival

**Variable**	**Cox proportional hazard model no frailty**			**Weibull model gamma frailty**		
	**Hazard ratio**	**95 % CI**	**P Value**	**Hazard ratio**	**CI**	**P Value**
**Infection (reference = No Infection)**						
**Sepsis**	1.36	1.20-1.54	<0.01	1.39	1.22-1.59	<0.01
**Sepsis x Year 1**	1.21	1.04-1.42	0.02	1.17	1.00-1.37	0.05
**Pneumonia**	1.55	1.42-1.70	<0.01	1.58	1.42-1.76	<0.01
**Pneumonia x Year 1**	1.15	1.02-1.30	0.02	1.11	0.98-1.25	0.11
**Sepsis & Pneumonia**	0.66	0.55-0.80	<0.01	0.66	0.55-0.80	<0.01
**Gender (reference = Male)**						
**Female**	0.89	0.85-0.94	<0.01	0.89	0.85-0.94	<0.01
**Race (reference = White)**						
**Black**	1.04	0.96-1.13	0.33	1.04	0.96-1.13	0.34
**Other**	0.96	0.84-1.09	0.49	0.95	0.84-1.08	0.46
**Age Group (reference < 50)**						
**50 to 64**	1.76	1.49-2.08	<0.01	1.75	1.48-2.07	<0.01
**65 to 74**	2.07	1.77-2.41	<0.01	2.06	1.76-2.41	<0.01
**75 to 84**	3.12	2.67-3.64	<0.01	3.10	2.64-3.64	<0.01
**85 to 94**	5.25	4.47-6.16	<0.01	5.21	4.36-6.21	<0.01
**95 and above**	8.38	6.68-10.52	<0.01	8.26	6.47-10.55	<0.01
**Insurance (reference = non Medicaid)**						
**Medicaid**	1.22	1.14-1.30	<0.01	1.22	1.14-1.30	<0.01
**Number of subjects**	17537			17537		
**Number of failures**	9694			9694		
**Log pseudolikelihood**	−89334.21			−26182.6		

Tests of the Cox proportional hazards assumption showed that the effects of sepsis and pneumonia were not proportional over time. Thus, interactions between sepsis and pneumonia and analysis time (distinguishing the first year after discharge from the rest of the study period) were incorporated into the model. These analyses controlled for healthcare-associated infections and full results are shown in Additional file 2: Table S2.

### Utilization models

Table 3 presents estimation results for the negative binomial models (see Additional file 2: Table S2 for full utilization models including CLABSI and VAP). We found that sepsis was significantly related to increased hospital admissions in years 1 and 2 (IR = 1.40 and 1.17, respectively) and emergency department visits in year 1 (IR = 1.28); however, these results were no longer significant once we accounted for utilization related to death (Model 2). Sepsis had a large and persistent relationship with long-term care utilization (IR ranged from 2.67 to 1.93 in years 1 through 5) and these associations were present in Model 2. Sepsis had a similar relationship with home healthcare utilization, with the exception of the first year post discharge. The relationship between sepsis and outpatient visits was in the opposite direction; that is, in Model 1 sepsis was related to decreased outpatient visits in year 1 (IR = 0.94, P < 0.05) and this relationship was present in Model 2 for years 1 and 2 (IR = 0.91 and 0.91, all P values less than 0.05).

**Table 3:** Utilization models for sepsis and pneumonia

	**IR**	**Sepsis**			**IR**	**Pneumonia**		
		**Model 1**	**IR**	**Model 2**		**Model 1**	**IR**	**Model 2**
		**P**		**P**		**P**		**P**
**Inpatient Admissions**								
Year 1	**1.40**	**<0.01**	1.07	0.12	**1.30**	**<0.01**	1.01	0.68
Year 2	**1.17**	**0.04**	1.06	0.42	**1.25**	**<0.01**	1.09	0.06
Year 3	1.07	0.50	0.97	0.67	**1.26**	**<0.01**	1.08	0.15
Year 4	0.95	0.64	0.92	0.40	**1.24**	**<0.01**	1.09	0.17
Year 5	1.00	0.99	0.89	0.21	1.12	0.17	1.11	0.20
**Outpatient Visits**								
Year 1	**0.94**	**0.05**	**0.91**	**0.01**	**0.95**	**0.03**	**0.94**	**0.01**
Year 2	0.92	0.07	**0.91**	**0.05**	0.96	0.24	**0.94**	**0.04**
Year 3	0.98	0.68	0.96	0.50	1.00	0.93	0.99	0.68
Year 4	0.95	0.39	0.97	0.55	0.94	0.13	0.94	0.11
Year 5	0.93	0.27	0.95	0.37	0.95	0.25	0.95	0.24
**Emergency Department Visits**								
Year 1	**1.28**	**<0.01**	0.97	0.50	**1.29**	**<0.01**	1.00	0.94
Year 2	1.13	0.15	1.01	0.85	**1.17**	**0.01**	0.99	0.84
Year 3	1.19	0.10	1.06	0.48	**1.18**	**0.02**	1.02	0.66
Year 4	0.98	0.87	0.97	0.74	1.10	0.21	0.93	0.20
Year 5	0.95	0.62	0.94	0.41	1.02	0.85	0.92	0.16
**Long-term Care Visits**								
Year 1	**2.67**	**<0.01**	**2.15**	**<0.01**	**2.16**	**<0.01**	**1.62**	**<0.01**
Year 2	**2.01**	**<0.01**	**2.29**	**<0.01**	**1.71**	**<0.01**	**1.50**	**<0.01**
Year 3	**1.81**	**<0.01**	**2.20**	**<0.01**	**1.60**	**<0.01**	1.24	0.09
Year 4	**1.63**	**0.01**	**1.86**	**<0.01**	**1.89**	**<0.01**	**1.50**	**0.01**
Year 5	**1.93**	**<0.01**	**1.97**	**<0.01**	1.14	0.49	1.16	0.41
**Home Care Visits**								
Year 1	1.26	0.33	1.41	0.22	1.33	0.09	1.35	0.08
Year 2	**2.15**	**0.01**	**2.02**	**0.02**	1.39	0.10	1.35	0.16
Year 3	**1.96**	**0.05**	**2.37**	**0.02**	**1.80**	**0.01**	**1.72**	**0.01**
Year 4	**2.22**	**0.01**	**2.74**	**<0.01**	1.54	0.07	1.49	0.10
Year 5	**2.27**	**0.02**	**2.51**	**0.01**	1.41	0.17	1.40	0.15

In Models 1, censoring for the amount of time at risk during year of death is treated as random. In Models 2, an additional polynomial was admitted to account for changes in utilization as a function of “time-until-death”. All models are negative binomials; standard errors are calculated with Huber-White sandwich estimators.

The relationships between pneumonia and healthcare utilization were similar to those of sepsis with a few notable differences. In Model 1, pneumonia was associated with increased hospital admissions until year 4 (IRs 1.24 to 1.30, all P values less than 0.01) and emergency department visits until year 3 (IRs 1.18 to 1.29, all P values less than 0.05); however, similar to sepsis, these associations were not significant in Models 2. Similar to sepsis, pneumonia had a strong relationship with long-term care admissions in year 1 (IR = 2.16, P < 0.01) and this persisted until year 4 (IR = 1.89, P < 0.01); these relationships were also significant in Model 2 for years 1, 2 and 4 (all P values less than 0.01). Pneumonia was also associated with increased home healthcare visits in year 3 (Model 1, IR = 1.80; Model 2 IR = 1.72, P values ≤ 0.01).

**Figure 2:**
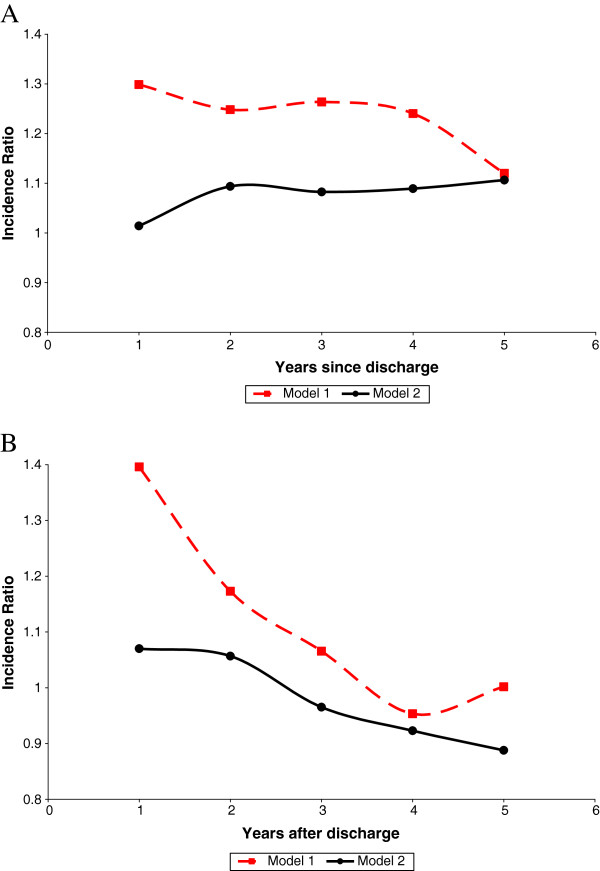
**Inpatient admission incidence ratios associated with infections.** Incidence ratios associated with (A) pneumonia and (B) sepsis are shown. Model 1 estimates the full relationship between infection and utilization but treats censoring (time at risk) as random. Model 2 separately identifies an underlying pattern of utilization following discharge and an additive component based on the time until death following discharge. It admits a polynomial to account for changes in utilization as a function of time until death.

The relationships between sepsis and pneumonia and inpatient admissions are illustrated in Figures 2A and 2B, respectively. These figures highlight the importance of increased mortality in the relationship between sepsis and inpatient admissions, and the modest direct association that remained after controlling for utilization related to death.

## Discussion

Previous estimates of the consequences associated with pneumonia and sepsis have focused mainly on immediate consequences of infection such as inpatient costs and short term mortality; and, previous investigators have not teased out the proportion of utilization associated with death. This rigorous econometric study used a large, comprehensive dataset to examine the relationships between these infections and five-year mortality and healthcare utilization. Furthermore, our specifications allowed us to examine the effects of infection on underlying utilization as well as the effects of infection on utilization related to death.

We found large and statistically significant increases in mortality associated with sepsis and pneumonia. Perhaps not surprisingly, these mortality effects were largest in the first year following an ICU stay in which the diagnosis of infection was made compared with subsequent years. Although the effects were persistent both for pneumonia and sepsis, they were larger over time for pneumonia. In our analyses, we used extensive controls for pre-admission comorbid conditions, conditional on being discharged alive from the index hospitalization, as well as models that accounted for unobserved frailty among subjects. We also controlled for other known measureable covariates such as gender, age and dual eligibility for Medicare and Medicaid. It may be that despite our state-of-the-art risk adjustment, those with pneumonia were frailer or it may be related to persistent physiologic changes associated with pneumonia. Yende et al. examined 1-year and 5-year mortality in 106 patients hospitalized with pneumonia and speculated that the infection triggers pathophysiologic processes that persist beyond recovery, possibly due to increased cytokines or persistent abnormalities in the innate immune response [8]. However, the mechanism underlying the larger, persistent long-term mortality effect related to pneumonia relative to sepsis is unclear.

We found significant increases in inpatient admissions in the first two years after infection for those with sepsis and for the first 4 years for those with pneumonia. This was due, however, to the association between infection and mortality. There was very little direct relationship between infection and downstream utilization after controlling for the time until death, suggesting that excess utilization manifested in those that eventually died; nevertheless, the indirect association between infection and healthcare utilization was large. Not surprisingly, we found very similar results with respect to emergency department visits.

The relationships between outpatient visits and infections were different from those of inpatient admissions and emergency department visits. We found no evidence of increases in outpatient admissions with or without controlling for the time until death. Indeed, we found limited evidence that outpatient visits were negatively associated with infections within 2 years of discharge from the index admission. There were, however, very large and lasting increases in long-term care admissions and home healthcare visits associated with sepsis and pneumonia, even after accounting for the role of death. These results are particularly important because of their magnitude and because of the high costs of long-term care. For example, individuals were approximately twice as likely to be admitted to long-term care in each of the five years following sepsis after controlling for utilization associated with death. These results, combined with those of the inpatient admissions models, indicate that infections have important long-term economic consequences in addition to the increased immediate costs.

We found no evidence that the long-term consequences of CLABSI differed from sepsis, nor evidence that the long-term consequences of VAP differed from pneumonia, either with respect to mortality or utilization. While these results may have important implications, they are based on relatively few VAP and CLABSI cases and may be imprecise. Previous research in this area is sparse. Similar to our results, in one other study with a relatively small sample size (178 hospital-associated methicillin-resistant *Staphylococcus aureus* [MRSA] bloodstream infections compared to 19 community-associated MRSA bloodstream infections) investigators found no differences in one year mortality between the two groups [29]. However, in another study, patients surviving discharge and categorized as having hospital-associated pneumonia (n = 66) were at greater risk of dying than those with community-associated pneumonia (n = 210) [13]. Clearly, more research is needed in this area, especially with the occurrence of an increasing number of community-associated infections as well as the national healthcare priority of reducing the high cost, high morbidity and mortality problem of healthcare associated infections [6,8,30].

This study has a number of strengths and limitations. The most important concern is that the associations may not be causal, but rather due to unobserved health status or severity of illness that is positively associated with the risk of infection *and* the outcomes (mortality and utilization). This concern is mitigated by several factors. First, the sample consisted only of elderly individuals who were admitted to an ICU, and our utilization models were estimated on a sample that included only those individuals who were discharged alive from the index ICU stay. Using a comparison group from the same population increases the homogeneity of the sample and ensures conservative findings (that is, a bias toward the null), particularly if there is a healthy survivor effect that reduces the frailty of those who survived among the infection groups versus those who survived among the controls. Second, we had access to a unique dataset that allowed us to follow elderly ICU patients over five years and obtain utilization data to develop state-of-the art health status controls for the period prior to the index hospitalization, and we admitted a variety of specifications of these health status controls into our models. Third, we estimated survival models that admitted unobserved frailty. Fourth, we are only studying those admitted to an ICU, and the long-term effects of those hospitalized with an infection compared to a community (non-hospitalized) control group may actually be greater; that is, our use of ICU patients without infections as controls is likely to bias the analysis towards the null representing a conservative estimate of these effects.

We found the results to be substantively unchanged under a variety of risk adjustment specifications, including ones that controlled for the full set of 184 hierarchical condition codes, and alternative specifications that included the number of hierarchical condition codes and aggregated condition codes of each subject. We also found little evidence of frailty in the survival models after admitting the hierarchical condition codes or aggregated condition codes health status controls. Nevertheless, though our results are suggestive, they should be viewed as associations, and causal interpretations should be made with caution.

Although we estimated models for a number of long-term healthcare utilization categories, our outcomes were limited to those measures available in the Medicare files. There were no data on patient out-of-pocket costs or quality of life. These data are needed to understand fully the burden of these infections from a societal perspective.

## Conclusions

We found that all infections studied had significant and lasting adverse consequences. Yet, many of these infections may be preventable. As we have identified the significant long-term health and healthcare utilization consequences of sepsis and pneumonia, these estimates should be included in future public health decision making in regards to investments in infection prevention interventions both in the community and hospitals.

## Abbreviations

CLABSI: Central-line-associated bloodstream infections; VAP: Ventilator-associated pneumonia; ICU: Intensive care unit; HR: Hazard ratios; IR: Incidence ratios; MRSA: Methicillin-resistant *Staphylococcus aureus*.

## Competing interests

The authors declare that they have no competing interests.

## Authors’ contributions

AD, EL, JZ and PS participated in the design of the study. EL, PS and MP participated in data acquisition and collection. AD and HL analyzed the data. AD and PS drafted the manuscript. All authors participated in data interpretation, and read and approved the final manuscript.

## Funding

This study was funded by the National Institute of Nursing Research (R01NR010107). The content is solely the responsibility of the authors and does not necessarily represent the official views of the National Institute of Nursing Research or the National Institutes of Health.

## Pre-publication history

The pre-publication history for this paper can be accessed here:

http://www.biomedcentral.com/1472-6963/12/432/prepub

## Supplementary Material

Additional file 1 Derivation of terms to characterize continuous time-until-death functions in discrete-time utilization models. Click here for file

Additional file 2 Full results including CLABSI and VAP. Click here for file
