# Supplementary material for: Long-term survival and healthcare utilization outcomes attributable to sepsis and pneumonia
Source: BMC Health Serv Res. 2012 Nov 26;12:432. doi: 10.1186/1472-6963-12-432 (PMC3534544; doi:10.1186/1472-6963-12-432)
Supplement: Additional file 1 — Derivation of terms to characterize continuous time-until-death functions in discrete-time utilization models. [file 1472-6963-12-432-S1.pdf]

Derivation of terms to characterize continuous time-until-death functions in discrete-time utilization models

Poisson and negative binomial models characterize event rates during discrete periods. The process generating the events, however, may be continuous in time. In that case, a period's event rate will depend on the integral of the continuous function from the beginning to the end of the period [1-3]. We make use of this fact to define covariates based on arbitrary continuous functions designed to model patterns of utilization prior to death. This is required because (1) utilization is affected by deteriorating health prior to death and (2) death does not conveniently occur at the end of the calendar year. The contribution to healthcare utilization of the impending death, therefore, will depend on when the death occurred within the last period and the shape of the function that captures the contribution of impending death on utilization. We model that contribution as a continuous (time until death) process, which we refer to as the "time-until-death" function, using several alternative functional forms. Note that our models have two time indices: one accumulating annual periods moving forward and indexing the discrete annual periods from discharge and one moving backwards measuring continuous time until death. We integrate the proposed time-until-death functions over the proper time intervals to identify the contribution of the time-until-death function during the annual period following discharge from the index ICU stay.

Consider a time-until-death function defined to be active during one year prior to death. Let t_d identify the year during which death occurred following discharge from the index ICU. Then t_d -1 is the year before the year of death. Let s be the fraction of the last year of life that occurred in year t_d-1 and 1-s be the fraction of the last year of life that occurred in the year of death (t_d). Consider a linear "time-until-death" function that additively increases utilization (in addition to an underlying pattern of utilization) from 0 precisely 12 months before death and

linearly increases until the time of death. In this case, only a subject's last two annual observations would be affected. If *a* is the slope of the linear time-until-death function, then the contribution of that function in t_d-1 is given by

$$\int_0^s f(t)dt = \int_0^s atdt = a\left(\frac{s^2}{2}\right).$$

In the year of death (t_d), the contribution of the linear time-until-death function is given by

$$\int_s^1 f(t)dt = \int_s^1 atdt = a\left(\frac{1-s^2}{2}\right).$$

Therefore, to admit a linear time-until-death function over the last 12 months of life, we define a single new measure with value $\left(\frac{s^2}{2}\right)$ in the year before death and $\left(\frac{1-s^2}{2}\right)$ in the year of death. The estimated coefficient of this measure is the death function slope parameter (*a*). Using a similar approach, we can identify the terms required to estimate a quadratic function in time until death. In the year before death, we have

$$\int_0^s f(t)dt = \int_0^s at + bt^2 dt = a\left(\frac{s^2}{2}\right) + b\left(\frac{s^3}{3}\right),$$

and in the year of death

$$\int_s^1 f(t)dt = \int_0^s at + bt^2 dt = a\left(\frac{1-s^2}{2}\right) + b\left(\frac{1-s^3}{3}\right).$$

In this case, then, we admit two additional measures, one to estimate the coefficient on the linear term (*a*) and one to estimate the coefficient on the quadratic term (*b*). The linear measure is defined as above. The quadratic measure takes the value $\left(\frac{s^3}{3}\right)$ in year t_d-1 and $\left(\frac{1-s^3}{3}\right)$ in year t_d.

The above can easily be generalized to alter the period during which the death function operates. Extending the period (>12 months) requires integrals over each of the last 3 years of life (or more).

This figure graphs the estimated “prior-to-death” effect specified as a 3rd order function and operative over 2 years prior to death. It shows a modest increase in inpatient admissions until the abrupt acceleration in the last six months of life.

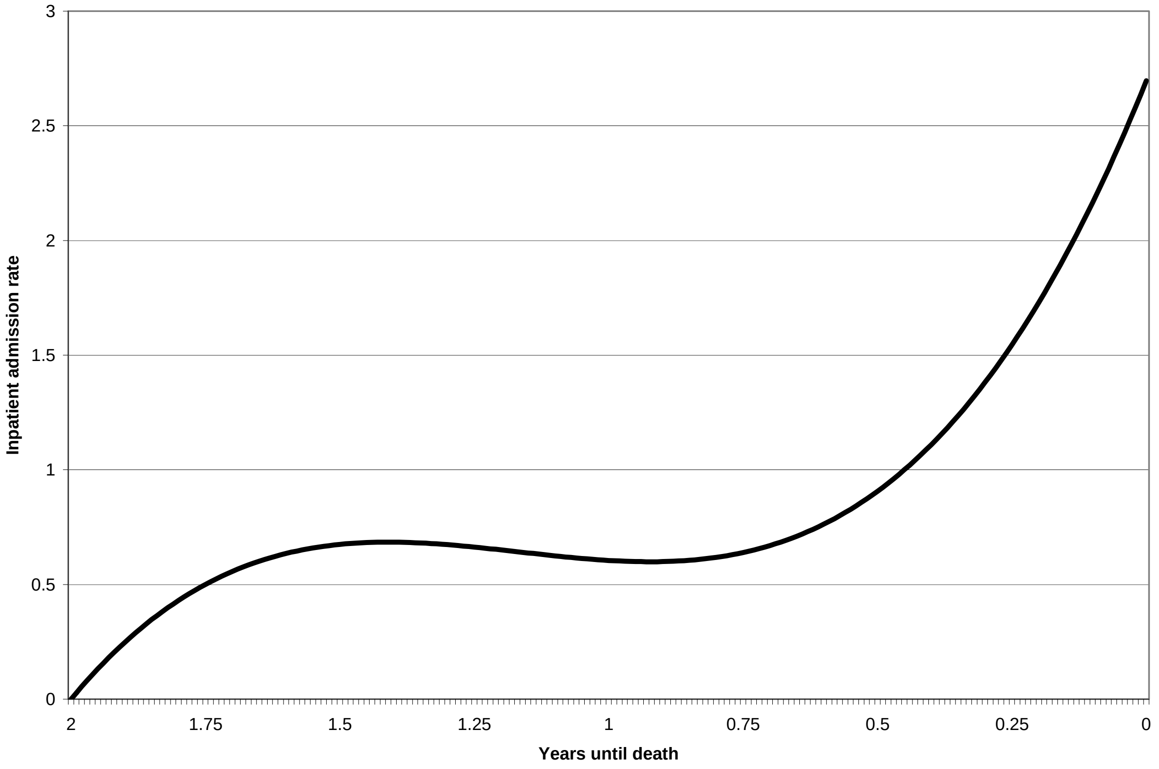

ADDITIONAL REFERENCES

1. Klein RW, Roberts SD: **A time-varying Poisson arrival process generator**. *Simulation* 1984, **43**(4):193-195.
2. Leemis LM: **Nonparametric estimation of the cumulative intensity function for a nonhomogeneous Poisson process**. *Manage Sci* 1991, **37**(7):886-900.
3. Arkin BL, Leemis LM: **Nonparametric estimation of the cumulative intensity function for a nonhomogeneous Poisson process from overlapping realizations**. *Manage Sci* 2000, **46**(7):989-998.
