# Supplementary material for: Long-term survival and healthcare utilization outcomes attributable to sepsis and pneumonia
Source: BMC Health Serv Res. 2012 Nov 26;12:432. doi: 10.1186/1472-6963-12-432 (PMC3534544; doi:10.1186/1472-6963-12-432)
Supplement: Additional file 2 — Full results including CLABSI and VAP. [file 1472-6963-12-432-S2.docx]

The Kaplan-Meier survivor functions in the figure below illustrate that the CLABSI and sepsis cohorts were neither substantively nor statistically different from one another and this was similar for the survivor functions of VAP and pneumonia. Table 1 shows that the relationships between CLABSI and VAP and healthcare utilization were not as pronounced as sepsis and pneumonia for most utilization categories. However, those with CLABSI did have greater use of LTC and those with VAP had greater use of HC (see Table 2). In both of these instances, the relationships persisted after controlling for utilization related to mortality.

**Figure Full Results of Kaplan-Meier Survivor Functions, including CLABSI and VAP**


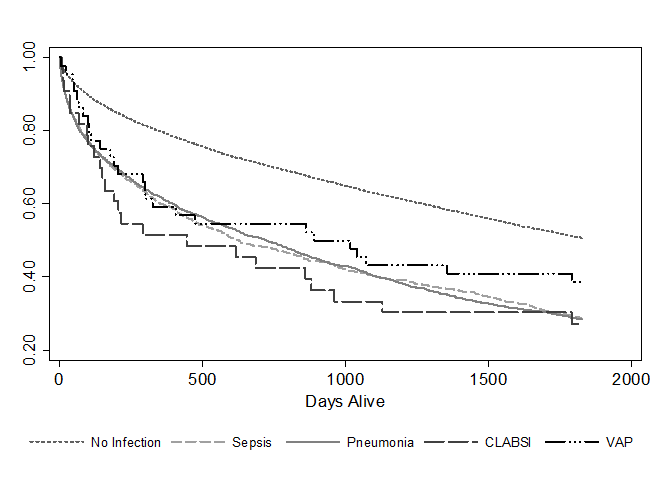


CLABSI – central-line associated blood stream infection

VAP – ventilator associated pneumonia

An overall log-rank test showed that the survivor functions differed across patient cohorts (P < 0.01). The survivor function of the control group was also longer than each infection group (P < 0.01 for sepsis, pneumonia and CLABSI and P = 0.03 for the VAP group); however, there were no significant differences between infection groups at the 5% level.

**Additional Table 1 Full Results of Cox Proportional Hazard and Frailty Models, including CLABSI and VAP**

|  | **Cox Proportional Hazard Model** | | | **Weibull Model** | | |
| --- | --- | --- | --- | --- | --- | --- |
|  | **No Frailty** | | | **Gamma Frailty** | | |
| **Variable** | **Hazard Ratio** | **95% CI** | **P Value** | **Hazard Ratio** | **95% CI** | **P Value** |
|  |  |  |  |  |  |  |
| **Infection (reference = No Infection)** |  |  |  |  |  |  |
| **CLABSI** | 1.66 | 0.91-3.01 | 0.10 | 1.67 | 0.92-3.04 | 0.09 |
| **VAP** | 1.65 | 1.15-2.35 | 0.01 | 1.66 | 1.17-2.37 | 0.01 |
| **CLABSI & VAP** | 0.39 | 0.13-1.15 | 0.09 | 0.38 | 0.13-1.15 | 0.09 |
| **Sepsis** | 1.36 | 1.20-1.54 | <0.01 | 1.39 | 1.22-1.59 | <0.01 |
| **Sepsis x Year 1** | 1.21 | 1.04-1.42 | 0.02 | 1.17 | 1.00-1.37 | 0.05 |
| **Pneumonia** | 1.55 | 1.42-1.70 | <0.01 | 1.58 | 1.42-1.76 | <0.01 |
| **Pneumonia x Year 1** | 1.15 | 1.02-1.30 | 0.02 | 1.11 | 0.98-1.25 | 0.11 |
| **Sepsis & Pneumonia** | 0.66 | 0.55-0.80 | <0.01 | 0.66 | 0.55-0.80 | <0.01 |
| **Gender (reference = Male)** |  |  |  |  |  |  |
| **Female** | 0.89 | 0.85-0.94 | <0.01 | 0.89 | 0.85-0.94 | <0.01 |
| **Race (reference = White)** |  |  |  |  |  |  |
| **Black** | 1.04 | 0.96-1.13 | 0.33 | 1.04 | 0.96-1.13 | 0.34 |
| **Other** | 0.96 | 0.84-1.09 | 0.49 | 0.95 | 0.84-1.08 | 0.46 |
| **Age Group (reference < 50)** |  |  |  |  |  |  |
| **50 to 64** | 1.76 | 1.49-2.08 | <0.01 | 1.75 | 1.48-2.07 | <0.01 |
| **65 to 74** | 2.07 | 1.77-2.41 | <0.01 | 2.06 | 1.76-2.41 | <0.01 |
| **75 to 84** | 3.12 | 2.67-3.64 | <0.01 | 3.10 | 2.64-3.64 | <0.01 |
| **85 to 94** | 5.25 | 4.47-6.16 | <0.01 | 5.21 | 4.36-6.21 | <0.01 |
| **95 and above** | 8.38 | 6.68-10.52 | <0.01 | 8.26 | 6.47-10.55 | <0.01 |
| **Insurance (reference = non Medicaid)** |  |  |  |  |  |  |
| **Medicaid** | 1.22 | 1.14-1.30 | <0.01 | 1.22 | 1.14-1.30 | <0.01 |
|  |  |  |  |  |  |  |
| **Number of subjects** | 17537 |  |  | 17537 |  |  |
| **Number of failures** | 9694 |  |  | 9694 |  |  |
| **Log pseudolikelihood** | -89334.21 |  |  | -26182.6 |  |  |

CLABSI – central-line associated blood stream infection

VAP – ventilator associated pneumonia

Tests of the Cox proportional hazards assumption showed that the effects of sepsis and pneumonia were not proportional over time. Thus, interactions between sepsis and pneumonia and analysis time (distinguishing the first year after discharge from the rest of the study period) were incorporated into the model.

**Additional Table 2 Full Utilization Models Inpatient Admissions, Outpatient Visits, ED Visits, Long-Term Care admissions, and Home Care Visits**

CLABSI – central-line associated blood stream infection; ED – Emergency Department; VAP – ventilator associated pneumonia
